# Data Centric Sensor Stream Reduction for Real-Time Applications in Wireless Sensor Networks

**DOI:** 10.3390/s91209666

**Published:** 2009-12-02

**Authors:** Andre Luiz Lins Aquino, Eduardo Freire Nakamura

**Affiliations:** 1 Computer Science Department, Federal University of Ouro Preto, Ouro Preto, MG, Brazil; 2 FUCAPI - Analysis, Research and Technological Innovation Center, Manaus, AM, Brazil; E-Mail: eduardo.nakamura@fucapi.br

**Keywords:** sensor-stream reduction algorithms, wireless sensor network, data-centric routing algorithm

## Abstract

This work presents a data-centric strategy to meet deadlines in soft real-time applications in wireless sensor networks. This strategy considers three main aspects: (i) The design of real-time application to obtain the minimum deadlines; (ii) An analytic model to estimate the ideal sample size used by data-reduction algorithms; and (iii) Two data-centric stream-based sampling algorithms to perform data reduction whenever necessary. Simulation results show that our data-centric strategies meet deadlines without loosing data representativeness.

## 1. Introduction

Despite their potential application, wireless sensor networks (WSNs) [1-3] have severe resource restrictions, such as low computational power, reduced bandwidth, and limited energy sources. Some applications are characterized by their emergency to deliver the data (real-time applications), *i.e.*, the data gathering has tight deadlines. Examples of these applications include: surveillance systems, biometric sensing, and intrusion detection. These applications have soft real-time characteristics, *i.e.*, environment is not controllable, applications usually use probabilistic models to process data, and communication does not have acknowledgment.

By considering the real-time applications in WSNs, we can identify some related work. In general, current contributions consider architectures and mathematical models for general applications [4–6]. Aquino *et al.* [7] propose and evaluate a design strategy to determine minimum deadlines used by a specific stream reduction algorithm in general WSNs applications. However, routing and application-level solutions for specific real-time scenarios have been recently proposed [8–10].

In WSNs applications, physical variables, such as temperature and luminosity, can be monitored continuously along the network operation. The data set representing these physical variables can be referred to as *data-stream* [11]—or *sensor-stream*, considering the WSNs context. As a consequence of this continuous monitoring, we might have high delays in such a way that real-time deadlines are not met. This motivation led us to propose a strategy to control the amount of data gathered by the network and its associated delay.

Before introducing our data-centric strategy, allow us to comment on data-stream related work. The data-stream contributions usually focus either in improving stream algorithms [12–15] or in applying the data-stream techniques to specific scenarios [16–20]. However, regarding data-stream solutions used in WSNs, we can identify a few researches that consider WSNs as distributed databases in which some functions (e.g., maximum, minimum and average) can be computed in a distributed fashion [21–25].

Considering real-time requirements and sensor-stream characteristics, we propose a data-centric strategy capable of reducing the data during data routing. In this case, the routing elements consider some application aspects, such as data type and deadline information. Our strategy considers: (1) a project design of real-time application to obtain the minimum deadlines; (2) an analytic model to estimate the ideal sample size used by the reduction algorithms; (3) and two stream-based sampling algorithms to perform data reduction when necessary during the routing task.

To validate our data-centric strategy, we use specific scenarios in which application deadlines cannot be met without data reduction. In our simulation, we use a naive tree routing based on shortest-path tree in a flat network. Application information is fed to relay nodes during build and rebuild tree phases. To identify the stream item delay, we consider that the clocks of the nodes are exactly synchronized. Thus, the time synchronization problem in WSNs [26] is not considered here. However, data quality is evaluated to show the associated data reduction impact. Simulation results show that our data-centric strategies meet deadlines without loosing data representativeness.

Regarding data reduction strategies for WSNs, current researches use data fusion, aggregation, compression or correlation techniques [3] to help save energy and reduce the packet delay [27–29]. The closer approach to sample stream reduction is the adaptive sampling, *i.e.*, the sampling strategy modifies following the phenomenons variations. The objective of this approach is to improve accuracy, identify correlation and eliminate redundancy [30–32]. However, there are some works that consider samples of different sources keeping the representativeness without overwriting the data, which can be applied to an uniform random or deterministic sample [16, 33–35]. It is important to highlight that our work considers the reduction of only one source, *i.e.*, our sampling is performed in each data set separately.

Our contribution can be highlighted through the analytical model, project design, different sample stream algorithm, and evaluation considering three more realistic real-time scenarios. The general contributions of our strategy are the following:

- **Data-stream:** In this work, we use sensor-stream algorithms as an in-network solution, and we improve the network performance by reducing the packet delay in real-time applications.
- **Data reduction:** Regarding data reduction, we show that we can meet real-time application deadlines when we use sensor-stream techniques during the routing task. This in-network approach represents a new contribution.
- **Real-time:** We present a analytical model to estimate the ideal amount of data-reduction, and we apply the stream-based solution in realistic real-time scenarios. To the best of our knowledge this is the first work that tries to quantify the reduction intensity based on real-time deadlines.

This work is organized as follows. Section 2. presents the data-centric real-time reduction problem. Section 3. shows how to design real-time sensor network applications by using stream-based data reduction. Section 4. discusses a formal formulation that is used to determine the ideal sample size. Section 5. describes the sampling stream reduction algorithms. Simulation results are presented in Section 6., and Section 7. presents our conclusions and outlook.

## 2. Problem Statement

The problem we address in this work is the sensor-stream reduction algorithms as a data-centric mechanism to meet deadlines in real-time applications. We consider the data-stream sampling technique to perform data reduction [36]. Since we use a sensor-stream algorithm for data reduction, the scope, and the problem itself can be defined as follows.

Let us consider a WSN monitoring physical or environmental conditions, such as temperature, sound, vibration, pressure, motion or pollutants, at different locations. Such a system is represented by the diagram [37]:


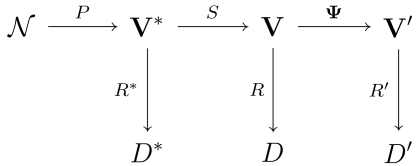


This diagram illustrates the following behavior:

- The ideal behavior denoted by *N* → V* → *D*, where *N* denotes the environment and the process to be measured, ***P*** is the phenomenon of interest, with V* their space-temporal domain. If complete and uncorrupted observation was possible, we could devise a set of ideal rules R* leading to ideal decisions D*.
- The sensed behavior is denoted by *N* → V* → V → *D*. In this case, we have a set of ***s*** sensors ***S=*(*S****_l_***,*…,S****_s_*), each one providing measurements of the phenomenon and producing a report in the domain ***V****_i_*, with **1 ≤ *i ≤ s***; all possible domain sets are denoted ***V=*(*V****_U_****…, V****_s_*). Using such information, we can conceive the set of rules *R* leading to the set of decisions *D*. We consider **V** to be a sensor-stream, due to its “time series” characteristics.
- The reduced behavior is denoted by *N* → V* → V → V → *D*′. Dealing with **V** may be too expensive in terms of, for instance, power, bandwidth, computer resources usage, and, specially, time delivery to meet the deadline requirements. Since the level of redundancy is not negligible in most situations, we can reduce this information volume. Sensor-stream reduction techniques are denoted by **Ψ**, and they transform the complete domain **V** into the smaller one **V**. New rules that use **V** are denoted by *R′*, and they lead to the set of decisions *D*′.

Based on these behaviors, the problem addressed in this work can be stated as follows:

### Problem definition

Given a sensor-stream behavior, how can we use a data-centric data reduction algorithm (**Ψ**) to meet application deadlines? Moreover, what is the impact over the decisions **D**, when we use the **Ψ** reduction over **V** generated by *S*?

**Figure 1.**
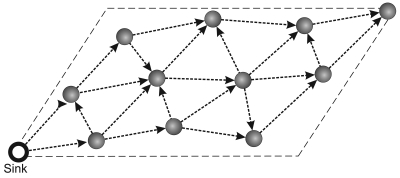

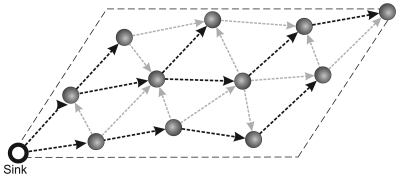

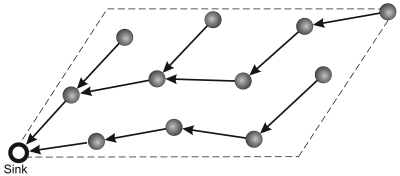
Build tree process.

To address the data-centric reduction problem in real-time applications, we consider the following assumptions:

- **WSN topology:** The set of sensors ***S =* (*S****_1_***,*…, S****_n_*) is distributed in a squared area ***A = L × L***. There is only one sink node located at (0, 0) on the left bottom corner. The density is kept constant and all nodes have the same hardware configuration.
- **Routing protocol:** The network communication is based on a multihop shortest-path tree [38] as the routing protocol. To evaluate only the data-centric stream reduction performance, the tree is built just once before the traffic starts and the network is kept static. The build tree process is depict in Figure 1. First in (a), the sink node sends a flooding message requesting to build tree. After this, in (b), the nodes sets your father node considering the first message received in flooding process (it is considered that the first packet received represents the shortest path to sink). Finally, in (c), we have the complete tree mounted.
- **Sensor-stream item: V***_i_* values are generated by one specific sensor located at (*L,L*) on the right top corner (the opposite side of sink node), for convention we use **V** to represent the stream generated. For each stream, we process one stream item **V = {V_1_,*…*, V***_n_***{** where the amount of data stored before the data sent is **| V| =** *N*. The generation is continuous at regular intervals (periods) of time. We consider gaussian data (*μ* **=** 0.5 and ***σ =*** 0.1) sent in bursts.
- **Quality of a sample:** To assess the impact of data reduction on data quality, based on decision *D*, we consider two rules: *R_dst_* and *R_val_*. The rule *R_dst_* aims at identifying whether **V** and **V′** data distributions are similar. To compute this distribution similarity (T), we use the Kolmogorov-Smirnov test [39]. The rule *R_val_* evaluates the discrepancy among the values in sampled streams, *i.e.*, if they still represent the original stream. To quantify this discrepancy (**Φ**), we compute the absolute value of the largest distance between the average value of the original data, and the lower or higher confidence interval values (95%) of the sampled data:
  $$\Phi =\max\{|\upsilon_{\mathrm{low}}-a\upsilon g_{g}|,|\upsilon_{\mathrm{hig}}-a\upsilon g_{g}|\}$$
  in which the pair (*v_low_*; *v_hig_*) is the confidence interval for the sampled data and *avg_g_* is the average (mean value) of original data [36]. These rules help us to identify the scenarios where our sampling algorithm is better than simple random sampling strategy.

These assumptions are considered in the whole paper. For instance, the routing algorithm is shortest path tree, the stream item is the set V = {Vi,…, V*_n_*{, and so on. In the next three sections, we answer the questions addressed in *Problem definition* by presenting the reduction design in real-time applications, the analytic model that estimate the ideal sample size |**V**′|, and the data-centric reduction algorithms.

### 3. Data-Centric Reduction Design in Real-Time WSNs Applications

**Figure 2.**
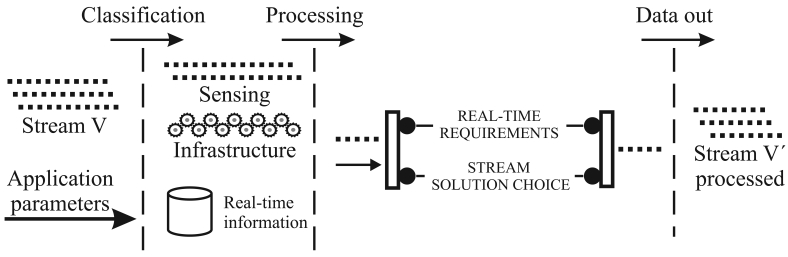
Data-centric reduction design in WSNs real-time application, the sensor view.

The first task of our data-centric strategy considers the design of real-time application. The objectives of this design are the: characterization of the stream flow while it passes by each sensor node; identification of the software components required by real-time applications by each sensor node; and identification of the required hardware resources by each sensor node. These aspects are illustrated in Figure 2, which shows the data-centric design in real-time WSNs applications, this design represents the sensor node view.

Basically, we have three steps to characterize the stream flow in each node: received data, data classification, and data processing. Considering the received data, **V** can be generated by the application or received from other nodes. In both cases, **V** is delivered to the routing layer. *Application parameters*, used to help the reduction phase, are also received whenever the routing tree is rebuilt. Once a sensor node receives **V**, we need to classify its type (classification step). In our case, the types considered are the *sensing* received from the application and the *infrastructure* received from other nodes. This classification is important because the routing layer behavior will be different for each one. When the node receives the *application parameters*, the *real-time information* database must be updated with new information. Such information include, for example, application deadlines, hops towards the sink, and time towards the sink. In the processing step, *real-time requirements* are verified. These requirements are used to decide the more suitable reduction strategy (*stream solution choice*), it is important highlight that this requirements are dynamically updated when the stream is received, *i.e.*, the relay node has only the local information. This occurs because the reduction may lead to different outputs with different “data qualities”. In our case, in this step, we determine **| V′|** according to the deadline requirements. The sample size determination and the reduction algorithm will be presented latter on. Finally, in the data out step, **V**, which may be reduced, is routed towards the sink.

In Figure 2, we can identify the software components required in real-time applications: a classification component to identify the type of **|V|**; an application-parameter component to process and store real-time application parameters; an oracle component to verify when the current stream item requires reduction; a stream size estimation component to compute **|V′|**, when necessary; a reduction component to perform the reduction; and a data out component to set the new parameters aggregated toV′.

Finally, the hardware resources necessary must be identified considering the **|V|** supported and the reduction algorithm complexity. **|V|** and **|V′|** are used to estimate the memory and bandwidth necessary to conceive the real-time WSN solution. In addition, the complexity of the reduction algorithm is important to determine the computational power necessary to apply the reduction strategy. These aspects are important to conceive a successful data-centric reduction for real-time applications.

### 4. What Is the Ideal Sample Size?

The second task of our data-centric strategy considers the **|V′|** estimation. The objectives of this estimation is to allow relay nodes to perform the reduction in a data-centric way, *i.e.*, the routing layer uses application information to meet real-time requirements by applying **Ψ** to reduce **|V|**.

In the *stream solution choice* of processing step (Figure 2) we determine **|V′|** necessary to meet the deadline specified in *real-time requirements*. In this case, to determine **|V′|**, every sensor node has a maximum packet size (***ps***) permitted. In our case, we consider ***p****_s_* **=** 20 items. However, every relay node knows its hop and time distances (considering only one packet) to the sink node, *h_dst_* and *t_dst_* respectively. This information is fed during the tree building phase, and stored in *real-time information* database.

In some cases, **V** needs to be fragmented in **V =** {**V^1^*…* V′**{, where *n_f_* is the number of fragments. All **V***^j^* (0 *< j ≤ n_f_*) encapsulate the application deadline (*d_a_*), number of fragments (*n_f_*), instant the fragment was generated (*t_gen_*), and its number of hops (*h_src_*). Thus, the relay node has all information about the stream item when it receives only one fragment.

Every relay node computes the new local deadline (*d_l_*), as depicted at the following:


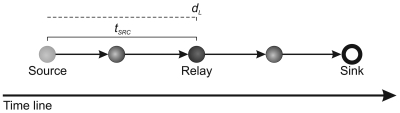


This deadline accounts the route between the relay and the sink node, and it is defined as

$$d_{l}=d_{a}-t_{\mathrm{src}}$$

where *t_src_* is the estimated time to deliver **V***^j^* from the source node to the current relay node,

$$t_{\mathrm{src}}=t_{\mathrm{now}}-t_{\mathrm{gen}}$$

Let us consider *t_src_* be the time of the V^1^ to travel from source at relay node. Then, **V**^2^ will arrive in *t_src_/h_src_* units of time (e.g., seconds), *i.e.*, estimated time for the last hop as depicted at the following:


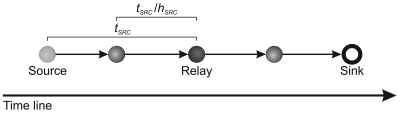


This consideration is necessary, because the information in the relay node is only about *t_src_*, therefore, the complete stream cames from the last relay node rather than directly from source. Thus, the estimated time receive **V** is

(1) $$t_{\mathrm{rec}}=(n_{f}-1)t_{\mathrm{src}}/h_{\mathrm{src}}$$

In a similar way, let us consider *t_dst_* the time of the **V**^1^ to travel from relay node at sink. Then, **V**^2^ will arrive in *t_dst_/h_dst_* units of time (e.g., seconds), *i.e.*, estimated time for the last hop as depicted at the following:


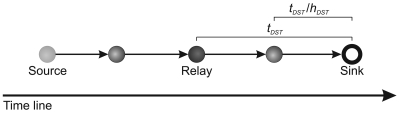


Thus, the estimated time to deliver **V** is

(2) $$t_{\mathrm{del}}=t_{\mathrm{dst}}+(n_{f}-1)t_{\mathrm{dst}}/h_{\mathrm{dst}}$$

The first term of the sum is considered in *t_del_* equation because **V**^1^ has not arrived yet. Remember that *t_dst_* and *h_dst_* are calculated when the tree is built. It is important highlighted that the transmissions between nodes in a WSN does not work like a pipeline. In our scenarios each sensor node has only one radio and it can either receive or send data, but not do both at the same time. So, the *t_rec_* and *t_del_* are estimated in each relay node separately.

Thus, |**V**′| is determined and used only if the *gap >* 0, *i.e.*, there is time to deliver the complete stream or part of it. *gap* is defined as

(3) $$\mathrm{gap}=d_{l}-\mathrm{delay}$$

where the stream *delay* at the sink node is

(4) $$\mathrm{dealy}=t_{\mathrm{rec}}+t_{\mathrm{del}}$$

The delay can be depicted as the following:


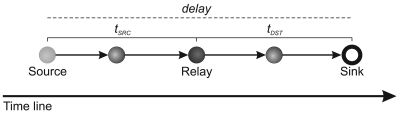


Thus, to compute |V′|, used to meet the application deadline, we consider the inequality

(5) gap>0

from (3) and (4) we have

$$d_{l}-\mathrm{delay}>0$$

$$d_{l}-(t_{\mathrm{rec}}+t_{\mathrm{del}})>0$$

0 using (1) and (2) we have

$$d_{l}-((n_{f}-1)t_{\mathrm{src}}/h_{\mathrm{src}}+t_{\mathrm{dst}}+(n_{f}-1)t_{\mathrm{dst}}/h_{\mathrm{dst}})>0$$

so that

$$n_{f}<1+\frac{h_{\mathrm{src}}h_{\mathrm{dst}}(d_{l}-t_{\mathrm{dst}})}{h_{\mathrm{dst}}t_{\mathrm{src}}+h_{\mathrm{src}}t_{\mathrm{dst}}}$$

considering that *n_f_ =* ⌈|V*′|/p_s_*⌉, we have

$$|\text{V}\prime |<p_{s}\left(1+\frac{h_{\mathrm{src}}h_{\mathrm{dst}}(d_{l}-t_{\mathrm{dst}})}{h_{\mathrm{dst}}t_{\mathrm{src}}+h_{\mathrm{src}}t_{\mathrm{dst}}}\right)$$

finally to reach the inequality we have

(6) $$|\text{V}\prime |=p_{s}\left(1+\frac{h_{\mathrm{src}}h_{\mathrm{dst}}(d_{l}-t_{\mathrm{dst}})}{h_{\mathrm{dst}}t_{\mathrm{src}}+h_{\mathrm{src}}t_{\mathrm{dst}}}\right)-1$$

Meanwhile, considering the |**V**′| presented by Aquino *et al.* [40], the sample size is estimated based on

(7) $$|\text{V}\prime |=p_{s}d_{a}/t_{\mathrm{dst}}$$

In order to identify both formulations, in simulation study (Section 6.), we will use the terms *complex formulation* and *simplified formulation* to represent Equations 6 and 7, respectively However, in both cases when the *gap ≤* 0 we consider |**V′**| = *ps* or the received is simple forwarded to preserve the data quality, because this *gap* means that the deadline was lost and the minor and more quickly data that can be delivered have to *p_s_* size.

### 5. Sensor-Stream Reduction

Finally, the third aspect of our data-centric strategy considers the sensor-stream reduction algorithm (**Ψ**). The objective of this reduction is to try to meet deadlines from real-time applications while keeping data fidelity (accuracy). The proposed sensor-stream reduction is motivated by the problem stated in Section 2. and evaluated in processing phase of project design described in Section 3.

The in-network (data-centric) reduction algorithm is integrated into a shortest-path routing tree. In this case, the routing tree is built, based on application requirements, from the sink (root) to the sources nodes by using a flooding strategy. In this flooding, *h_dst_* and *t_dst_* values are delivered to every sensor node. Once the routing tree is built, the source node can send **V** towards the sink. At this moment, relay nodes receive {V^1^*…* V*^nf^*{ in some packets (fragments) and forward them to another relay node until the sink is reached.

In this forward process, when a relay node receives **V**^1^, it checks the stream reduction criterion, in our case if *gap >* 0 (Equation 5). If the criterion is satisfied **V**^1^ is stored and the node waits to receive and store {**V^2^***^…^* **V***^nf^*{. Otherwise, all fragments are forwarded. It is important highlighted that this forwarding is in routing layer, considering the sensor radio, certainly, the fragments are queued until the sender radio turns able. Based on the *real-time information, |***V**′| is computed through Equation 6. When all fragments arrive, the **Ψ** reduction is enabled. This forwarding process is shown in Algorithm 1.

**Algorithm 1:** Pseudo-code of reduction decision.**Data:** V*^j^* – fragment stream received1**begin**2 “Get from V*^j^* the fragments information”3 **if***j* = 1 **then**4  “*gap* is computed through equations (2–4)”5  **if***gap >* 0 **then**6   “Enable **V** storage”_7_   
$|\text{V}\prime |=p_{s}\left(1+\frac{h_{\mathrm{src}}h_{\mathrm{dst}}(d_{l}-t_{\mathrm{dst}})}{h_{\mathrm{dst}}t_{\mathrm{src}}+h_{\mathrm{src}}t_{\mathrm{dst}}}\right)-1\{\text{Equation}\;6\}$8  **end**9 **end**10 **if***Storage is enabled***then**11  “Store **V***^j^*”12  **if***j* = *n_f_* then13   **V***←* “Compute **Ψ** on **V** with | **V′**| size”14   “Send**V**”15  **end**16 **end**17 **else**18  “Forward **V***^j^*”19 **end**20**end**

**Figure 3.**
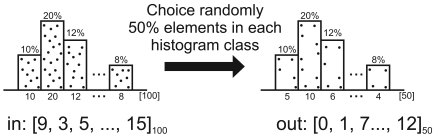

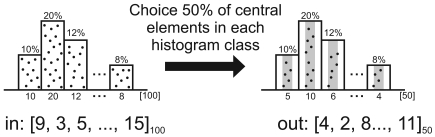
Reduction algorithms.

When the reduction is able, a histogram of **V** is built (line 13 of Algorithm 1). We consider a simple histogram, all elements sensed are between [0; 1] and we have 10 equals histogram classes. To obtain such a sample, we choose the central elements of each histogram class, respecting the sample size | V′| and the class frequencies of the histogram. Thus, the resulting sample will be represented by the same histogram. Meanwhile, considering the sensor-stream reduction algorithm presented by Aquino *et al* [40], the sample elements are randomly chosen in each histogram class instead of considering the central elements. The reduction algorithms, used here, and their operations are depicted in Figure 3. The random sampling (a), we have a “stream in **V**” with 100 elements, |**V**′| → 50% of **V** is randomly chosen (this choice is performed in each histogram class), and then a “stream out **V**′” is generated with | **V**′| = 50. The central sampling (b), we have again a “stream in **V**” with 100 elements, | **V′**| → 50% of **V** is choice considering the central histogram classes elements, and then a “stream out **V**′” is generated with |**V**′| = 50.

In order to identify both algorithms, in simulation study (Section 6.), we use **Ψ**_central_ and **Ψ**_random_ to represent the central and random elements choice, respectively. The **Ψ**_central_ sample reduction process is present in Algorithm 2.

Analyzing the Algorithm 2 we have:

#### Line 2

Executes in *O*(|**V**| log |**V**|);

#### Lines 11-15

Define the inner loop that determines the number of elements at each histogram class of the resulting sample, considering *H_cn_* as the number of histogram class and *n'_coli_* as the columns in sampled histograms, where 0 *< i < H_cn_*. The $\sum_{i}^{H_{cn}}{n^{\prime }}_{{\text{col}}_{i}}=\left|V^{\prime }\right|$, we have that this inner loop executes in *O*(|**V**′|) steps.

#### Lines 7-20

Define the outer loop in which the input data is read and the sample elements are chosen. Because the inner loop is executed only when condition in line 8 is satisfied, the overall complexity of the outer loop is *O*(|**V**|) + *O*(|**V**′|) = *O*(|**V**| + |**V**′|), since we have an interleaved execution. Let *n_col_* be the columns in original histograms, where 0 *< i < H_cn_*. Basically, before evaluating the condition of Line 8, *n_coli_* is accounted and |**V***|/H_cn_* interactions are executed. Whenever this condition is satisfied, *n'_coli._* is built and |V*′|/H_cn_* interactions are executed (Lines 11-15). In order to build the complete histogram, we must cover all classes (*H_cn_*), then we have *H_cn_* (|V| + |V′|)*/H_cn_*=|**V**| + |**V**′|.

#### Line 21

Re-sorts the sample in *O*(|**V′**| log |**V′**|).

Thus, the overall complexity is

*O*(|**V**| log |**V**|) + *O*(|**V**| + |**V**′|) + *O*(|**V**′| log |**V**′|) = *O*(|**V**| log |**V**|)

since |**V**′*| ≤ |***V**|. The sorting step is necessary, because, in our case, to build the histogram, we need the elements to be sorted, so that we always get the correct elements of **V′**. The space complexity is *O*(|**V**| + |**V**′|) = *O*(|**V**|) because we store the original sensor-stream and the resulting sample. Since every source node sends its sample stream towards the sink, the communication complexity is *O*(| **V**′*| D*), where *D* is the largest route (in hops) in the network.

Algorithm 2: Pseudo-code of **Ψ**_central_ sampling reduction.**Data:** V – original sensor-stream**Data:** |V′| - resulting sample size**Result:** V - resulting sample set1**begin**2 *Sort*(**V**)3 *wid ←* “Histogram's class width”4 *fst←0* {first index of histogram class}5 *n_col_ ←* 0 {number of elements per columns in V}6 *w←0**7* **for***k←*0 *to|***V**|-1do8  **if V**[k] *>***V** [*fst*]*+wid or k = |***V**|- 1 **then***9*   *n'_col_* ← ⌈n'_col_ | **V**′*|/|***V**| ⌉{number of elements per columns in V′}10   *index ←f st*+⌈(*n_col_-n'_col_*)*/2*⌉11   **for***l←*0 *to n'_col_***do**12    **V***′*[*w*] *←***V**[*index*]13    *w ← w* + 114    *index ← nextIndex*15   **end**16   *n_col_ ←* 017   *fst ← k*18  **end**19  *n_col_ ← n_col_* + 120 **end**21 *Sort*(**V′**) {according to the original order}22**end**

### 6. Simulation

This section presents the simulation study of our data-centric strategy in specific scenarios. We perform our evaluation by using the NS-2 (Network Simulator 2), version 2.33 (http://nsnam.isi.edu/nsnam/index.php/Main_Page). Each simulated scenario was executed with 33 random topologies. At the end, for each scenario we plot mean values with 95% (symmetric asymptotic) confidence intervals.

To identify assess the network behavior, we variate the number of nodes and the stream size (| **V**|). The evaluated parameters are the delay and error in data quality, specified by two statistical tests. The default parameters used in simulations are presented in Table 1.

**Figure 4.**
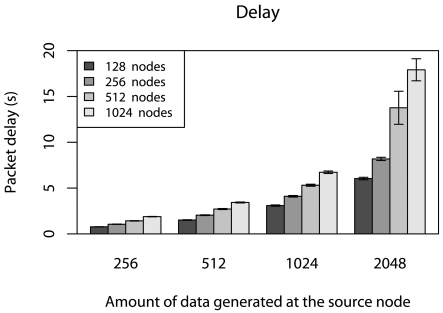
Minimum deadlines.

To evaluate the delay in real-time scenarios, it is important to determine the minimum deadline (*d_mm_*) for each number of nodes and |**V**| being considered. To do this, we consider different network sizes (128, 256, 512, and 1024), the |**V**| = {256, 512,1024, 2048{, and only one data source generating **V**. In our case, the *d_min_*values is determined by measuring the time between the first data packet sent by the source and the last packet received by the sink, *i.e.*, the time for **V** to be entirely received by the sink. Figure 4 illustrates *d_min_*values for all scenarios being considered.

It is important to highlight that if either application has a deadline smaller than the one shown in Figure 4 or the network is not in perfect conditions, then all data cannot be transmitted unless some reduction is performed. However, despite all nodes (sources) know the real time requirements of the packets they generate, they cannot infer the necessary data reduction locally because the network has some global restriction not perceived for them.

In the problem scope defined in Section 2., we discuss the impact of the solutions regarding the data quality, which is considered as our decision *D*. To assess the impact of data reduction on data quality, based on decision *D*, we consider the rules *R_dst_* and *R_val_* defined before. These rules are represented by T and **Φ** errors, respectively.

It is possible to apply these rules, because we consider the reduction of only one source, *i.e.*, our sampling is performed in each data set separately. For example, considering a simple network of nodes connected as a tree as *Sink-Node_A_-Node_B_*. After in network data reduction we give equal opportunity to the data points *of Node_A_* and *Node_B_* separately. So, clearly the data gathered in the sink will represent the original data of the network, where neither data nodes will be over-represented.

The deadlines for the real-time scenarios, that we consider, are 50% of the minimum deadlines with(out) concurrent traffic; minimum deadlines with delay caused by relay nodes in each packet transmitted with(out) concurrent traffic. These study are discussed in the next subsections. For all scenarios, we evaluate the simplified and complex formulations, both using **Ψ**_central_ or **Ψ**_random_. We use a Monte Carlo simulation [41] considering a complete mapping between the number of nodes and the stream size, only unusual results will be presented. However, the y-axis scale is not kept constant in all figures. The reason is that such a re-scale allow us to make better analysis.

#### 6.1. Half of Deadlines without Concurrent Traffic

**Figure 5.**
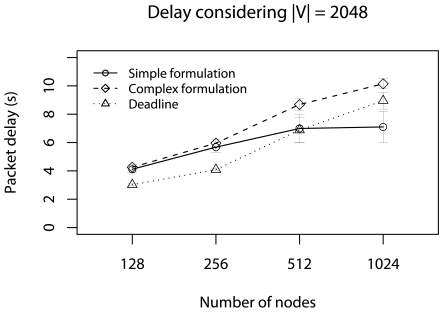
Delay considering the half of deadlines without concurrent traffic.

The first scenario considers half of minimum deadlines (*d_a_ = d_min_/2*) without concurrent traffic in this case, the application cannot send **V** and meet *d_a_*. In this case, **V** is reduced by using our data-centric strategy. In Monte Carlo simulation, we change the number of nodes (128, 256, 512, and 1024) and |**V**| (256, 512, 1024, and 2048). Figure 5 shows the delay results varying the number of nodes with |**V**| = 2048.

In this case, the *d_a_* cannot be met in many cases. However, without the our data-centric reduction strategy these delays would be even larger. When the number of nodes is 512 and 1024, the simplified formulation presents a smaller delay compared with the complex formulation, and the deadline is met.

The reason is that, in the simplified formulation, the reduction is harder and less data is forwarded. When the number of nodes is 1024, the simplified formulation delivers 19% of data, while the complex formulation delivers 25%. This indicates that, considering only the deadline achievement, the simplified formulation is more appropriate. However, the reduction ratio is greater.

**Figure 6.**
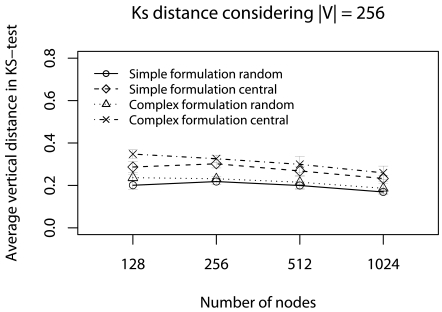
ϒ-error considering the half of deadlines without concurrent traffic.

**Figure 7.**
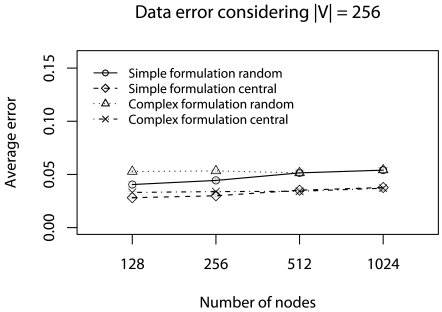
Φ-error considering the half of deadlines without concurrent traffic.

Regarding the data error evaluation. Figures 6 and 7 show the error evaluation for different numbers of nodes with |**V**| = 256. Figure 6 shows that in all cases we have T *≤* 40%. **Ψ**_random_, in both formulations, has a smaller ϒ-error because the random choice improves data dispersion, and the simplified formulation has a smaller ϒ-error.

Figure 7 shows that in all cases we have **Φ** ≈ 5%. Ψ_central_, in both formulation, has a smaller **Φ**-error because the central elements choice improves the average test. Again, the simplified formulation has a smaller **Φ**-error The error evaluation indicates that the simplified formulation is more appropriate in this specific scenario. Considering the evaluated algorithms, **Ψ**_random_ may be used when *R_dst_* is the rule priority, otherwise, **Ψ**_central_ should be used.

The partial conclusion, considering this critical real-time scenarios, is that the simplified formulation is more appropriate, because the deadlines are usually met while keeping data representativeness. Considering the sampling algorithm, **Ψ**_random_ or **Ψ**_central_ can be used when data application decisions are related, *R_dst_* or *R_val_*, respectively.

#### 6.2. Delay Caused by Relay Nodes without Concurrent Traffic

The second scenario, considers *d_a_ = d_min_* and all relay nodes delaying the stream fragments (**V**^j^)per *d_a_/*10000 or 0.01% of the *d_a_*. In this scenario, the application cannot send **V**, because the relay nodes eventually can be executing another task, consequently, the deadline *d_a_* cannot be met. We change the number of nodes (128, 256, 512, and 1024) and |**V**| (256, 512, 1024, and 2048). The objective of this scenarios is to identify the appropriate strategy when relay nodes have other high priority tasks.

**Figure 8.**
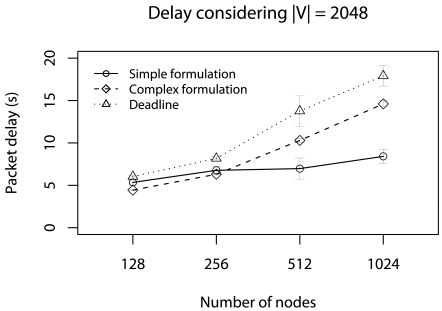
Delay considering the delay caused by relay nodes without concurrent traffic.

Figure 8 shows the delay results when we change the number of nodes with | **V**| = 2048. In contrast to prior scenario, *d_a_* is always met. The complex formulation, in a more realistic scenario, presents a better time usage, *i.e.*, the delay is closer to deadline values. This occurs because the **Ψ**-reduction estimation in sample case [Equation 7] is related to *d_a_, i.e.*, if *d_a_ > d_min_* reduction cannot be efficient, when we consider other delay aspects like concurrent traffic. Another important observation is that more data is received in the complex formulation strategy. When the number of nodes is 1024, nearly 20% of data are deliver, in the complex formulation, against 18%, in the simplified one. This fact indicates that, considering only the deadline achievement, the complex formulation is more appropriate to more realistic scenarios.

**Figure 9.**
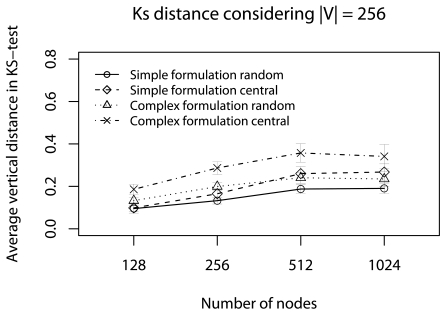
ϒ-error considering the delay caused by relay nodes without concurrent traffic.

**Figure 10.**
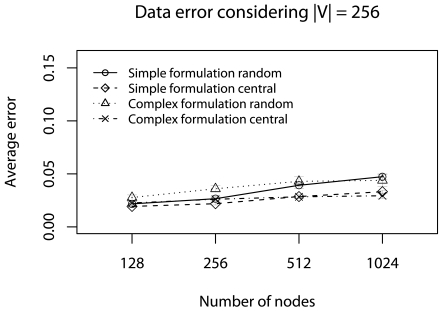
**Φ**-error considering the delay caused by relay nodes without concurrent traffic.

Figures 9 and 10 show the error evaluation when we vary the number of nodes and keep | V| = 256. Similar to previous scenario, the results (Figure 9) show that in all cases we have T ≤ 40%. **Ψ**_random_, in both formulation, has a smaller ϒ-error, because the random choice improves data dispersion. However, the simplified formulation has a smaller ϒ-error. In Figure 10, we always have **Φ** ≈ 5%. Again, **Ψ**_central_, in both formulation, has a smaller **Φ**-error ecause the central elements choice improves the average test. Although, the simplified formulation has a smaller **Φ**-error with 128, 256 and 512 nodes, the complex formulation presents a better performance for 1024 nodes.

Error evaluations suggest that the simplified formulation is more appropriate for small networks and the complex formulation is more scalable. In general, **Ψ**_random_ is preferable when the *R_dst_* has higher priority compared to the *R_val_*, otherwise, **Ψ**_central_ should be chosen. However, the complex formulation is more appropriate when we have large scale networks. The partial conclusion, considering more realistic real-time scenarios, is that the complex formulation is more appropriate, because the deadlines are met in all cases and the data representativeness is kept.

#### 6.3. Half of Deadlines with Concurrent Traffic

This scenario considers 50% of minimum deadlines (*d_a_* = *d_min_/*2) with concurrent traffic. We use a 128-node network (we do not consider more nodes due to NS limitations), and vary the percentage of nodes generating data traffic (16%, 20%, 25%, and 33% of 128 nodes) and |V| (256, 512, 1024, and 2048). The objective of this scenario is to identify the best strategy in critical applications when the network traffic gradually increases.

**Figure 11.**
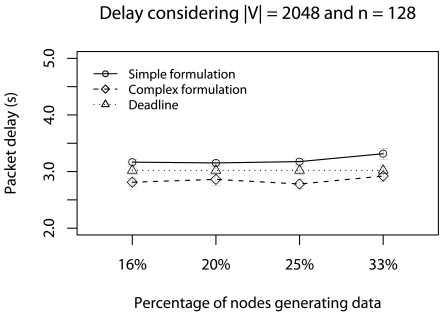
Delay considering the half of deadlines with concurrent traffic.

In Figure 11, *d_a_* is met only by complex formulation. The reason is that the Ψ-reduction, in complex formulation, is gradually performed during the data routing, and fewer data is delivered. Particularly, when the percentage of nodes generating data is 33%, nearly 6.5% of data is delivered by the complex formulation, while 10% is delivered by the simplified formulation. Thus, considering the deadline achievement, the complex formulation is more appropriate in this scenario.

**Figure 12.**
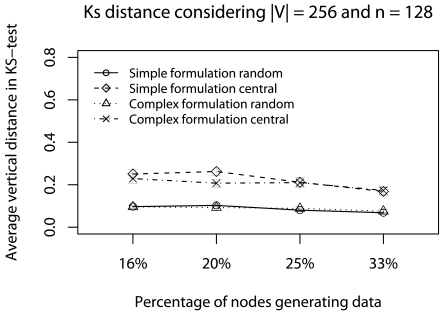
ϒ-error considering the half of deadlines with concurrent traffic.

**Figure 13.**
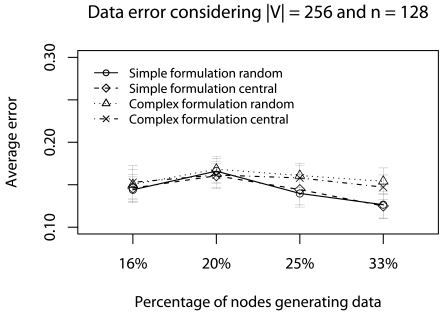
ϒ-error considering the half of deadlines with concurrent traffic.

Figures 12 and 13 show the error evaluation considering a 128-node network, varying the percentage of nodes that generate data traffic, and keeping |**V**| = 256. As we can see in Figure 12, results show that in all cases we have T ≤ 30%. In both formulations, **Ψ**_random_ has a smaller ϒ-error. However, in the complex formulation, **Ψ**_central_ presents a smaller ϒ-error when we have fewer data traffic (16% and 20%). The reason is that the complex formulation executes fewer consecutive **Ψ**-reductions. Figure 13 shows that, in all cases, we have **Φ** ≈ 20%, note the increase in terror, compared to previous scenarios (**Φ** ≈ 5%). The reason is that in both formulations there more consecutive **Ψ**-reductions affecting data quality. Therefore, considering the confidence interval, both **Ψ**-reductions have the same behavior. Again, when the percentage of nodes generating data is high, the simplified formulation has a smaller **Φ**-error.

The data error evaluation suggests that the simplified formulation is slightly better than the complex one. However, the partial conclusion, considering this critical and realistic real-time scenario, is that the complex formulation is more appropriate, because deadlines are met and data representativeness is kept. Considering the sampling algorithms, the behavior is kept in both **Ψ**-reduction strategies.

#### 6.4. Delay Caused by Relay Nodes with Concurrent Traffic

The last scenario considers *d_a_* = *d_min_*, concurrent traffic, and all relay nodes delay the stream fragments at 0.01% of the *d_a_*. Again, we use a 128-node network and vary the percentage of nodes generating traffic (16%, 20%, 25%, and 33% of 128 nodes) and |**V**| (256, 512, 1024, and 2048). The

objective of this scenario is to identify the best strategy when the relay nodes have extra tasks with high priority and the network has a traffic that gradually increases.

**Figure 14.**
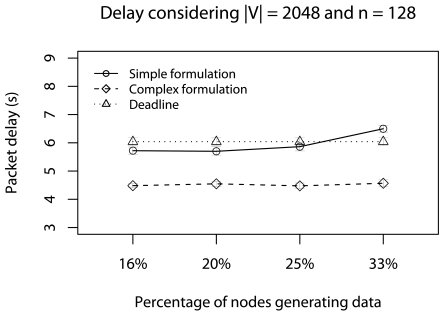
Delay considering the delay caused by relay nodes with concurrent traffic.

Figure 14 shows the delay results in a 128-node network varying the percentage of nodes generating traffic and |V| = 2048. The *d_a_* is met in most cases. The complex formulation presents a more scalable behavior considering the percentage of nodes generating data. This occurs because the **Ψ**-reduction estimation [Equation 7] is related to *d_a_*, *i.e.*, if *d_a_ ≥ d_min_* the **Ψ**-reduction cannot be efficient, when we consider other delay aspects, like concurrent traffic.

**Figure 15.**
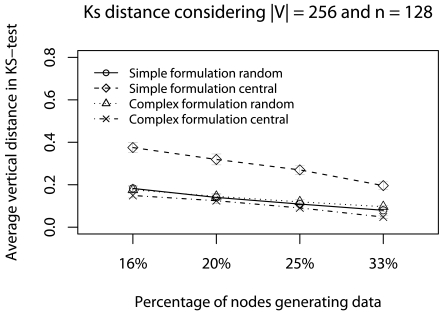
ϒ-error considering the delay caused by relay nodes with concurrent traffic.

**Figure 16.**
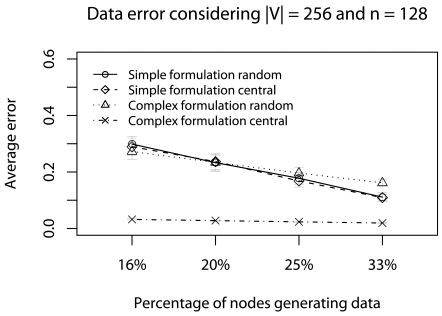
**Φ**-terror considering the delay caused by relay nodes with concurrent traffic.

Figures 15 and 16 show the error evaluations considering a network with 128-nodes, varying the percentage of nodes generating traffic and |**V**| = 256. Simplified and complex formulation are presented by using **Ψ**_central_ and **Ψ**_random_. Figure 15 shows that in all cases we have T *≤* 40%. The Ψ*_central_, in complex formulation, has a smaller ϒ-error. The reason is that the complex formulation performs the maximum Ψ-reduction sooner (*_central_ is executed once or twice). This result shows that because fewer successive *-reductions are performed, more representativeness is kept in the reduced data, *i.e.*, data degradation is mitigated.

Figure 16 shows that, in general, we have **Φ** *≤* 30%. The complex formulation with **Ψ**_central_ presents **Φ** *≈* 5%. The reason is that the **Ψ**_central_ has a smaller **Φ**-error because the central elements choice improves the average test and the complex formulation performs the maximum **Ψ**-reduction early.

The errors evaluation suggest that the complex formulation is more appropriate with the **Ψ**_central_ strategy. The partial conclusion, considering this scenario, is that the complex formulation is actually more appropriate, because the deadlines are met in all cases while keeping data representativeness. Considering the sampling algorithm, the **Ψ**_central_ strategy with complex formulation is always indicated.

### 7. Conclusions

In real-time applications of wireless sensor networks, the time used to deliver sensor-streams from source to sink nodes is a major concern. The amount of data in transit through these constrained networks has a great impact on the delay. In this work, we presented a data-centric strategy to meet deadlines in soft real-time applications for wireless sensor networks. This work represents shows how to deal with time constraints at lower network levels in a data-centric way.

With our data-centric strategy we met application deadlines in several scenarios. In additional, we showed how to design real-time sensor-stream reduction applications and a analytical model used to found the ideal sample size. Results showed the efficiency of the strategy by reducing the delay without losing data representativeness. If the application is not strongly dependent on data accuracy, or the network operates in exception situation (e.g., few resources remaining or urgent situation detection), then data reduction algorithms are powerful tool for real-time applications for resource-constrained networks.

As future work, we intend to match the proposed application-level solution with lower-level ones, for example, by considering some real-time-enabled signal processing method. In this case, not only data from a source is reduced, but similar data from different sources is also reduced, resulting in a more efficient solution. Another future work is to use feedback information to enable the source nodes to perform the reduction sooner. However, we intend to improve the central sampling algorithm complexity to *O*(*n*), by considering some rank selection algorithms.

## Figures and Tables

**Table 1.** Simulation parameters.

**Parameter**	**Values**
Network size	Varied with density
Queue size	Varied with stream
Simulation time (seconds)	1100
Stream periodicity (seconds)	10
Radio range (meters)	50
Bandwidth (kbps)	250
Initial energy (Joules)	1000
